# Acute Pancreatitis Induced by Methimazole in a Patient With Subclinical Hyperthyroidism

**DOI:** 10.1177/2324709615592229

**Published:** 2015-06-24

**Authors:** Katrina Agito, Andrea Manni

**Affiliations:** 1 Penn State University/Milton S. Hershey Medical Center, Hershey, PA, USA

**Keywords:** adverse drug reactions, drug-induced pancreatitis, methimazole, pancreatitis, hyperthyroidism

## Abstract

We report here a unique case of methimazole (MMI)-induced pancreatitis. To our knowledge, this is the sixth case reported in the literature and the first diagnosed in a patient with toxic multinodular goiter. A 51-year-old Caucasian female with a history of benign multinodular goiter and subclinical hyperthyroidism was started on MMI 10 mg orally daily. Three weeks later, she developed sharp epigastric pain, diarrhea, lack of appetite, and fever. Her lipase was elevated 5 times the upper limit of normal, consistent with acute pancreatitis. There was no history of hypertriglyceridemia, or alcohol abuse. Abdominal computed tomography was consistent with acute uncomplicated pancreatitis, without evidence of gallstones or tumors. MMI was discontinued, and her hyperthyroid symptoms were managed with propranolol. Her acute episode of pancreatitis quickly resolved clinically and biochemically. One year later, she redeveloped mild clinical symptoms of hyperthyroidism with biochemical evidence of subclinical hyperthyroidism. MMI 10 mg orally daily was restarted. Five days later, she experienced progressive abdominal discomfort. Her lipase was elevated 12 times the upper limit of normal, and the abdominal computed tomography was again compatible with acute uncomplicated pancreatitis. MMI was again discontinued, which was followed by rapid resolution of her pancreatitis. The patient is currently considering undergoing definitive therapy with radioactive iodine ablation. Our case as well as previous case reports in the literature should raise awareness about the possibility of pancreatitis in subjects treated with MMI in the presence of suggestive symptoms. If the diagnosis is confirmed by elevated pancreatic enzymes, the drug should be discontinued.

## Introduction

We report here a patient who experienced 2 acute episodes of pancreatitis both shortly following administration of methimazole (MMI), the first after the initial exposure and the second after rechallenge, with quick resolution of pancreatitis after MMI withdrawal in both cases. So far, only 5 cases of acute pancreatitis following MMI treatment have been reported worldwide. To our knowledge, this is the sixth case, and the first occurring in a patient with toxic multinodular goiter (MNG).

## Case Report

**Figure 1.**
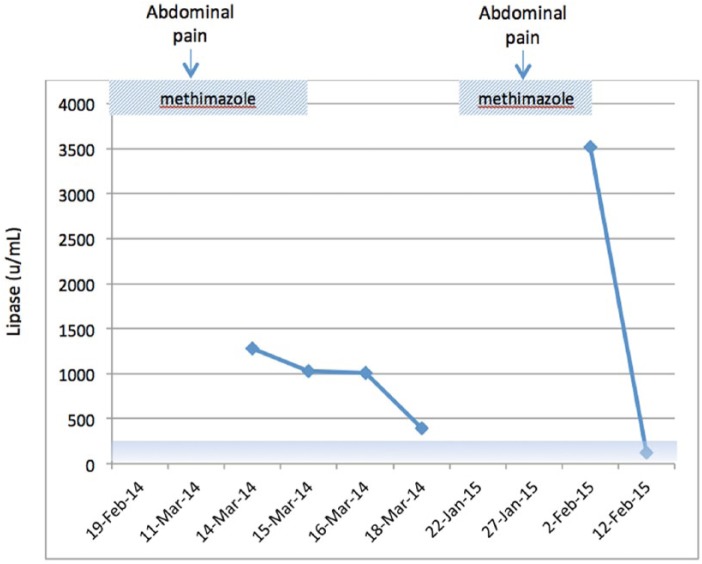
Profile of serum lipase on and off methimazole treatment in our patient. Shaded area represents normal range for serum lipase.

A 51-year-old Caucasian female with a history of biopsy-proven benign MNG presented with anxiety and shakiness. She had no signs of ophthalmopathy or pretibial myxedema. Her thyroid stimulating hormone was suppressed at 0.16 (0.45-4.1 µIU/mL), free T4 was normal at 1.27 (0.73-1.95 ng/dL), and free T3 was normal at 3.2 (2.5-5.3 pg/mL). Thyroid-stimulating immunoglobulin, antithyroid peroxidase, and thyroglobulin antibodies were negative. Her liver function tests (LFTs) were normal, including total bilirubin at 1.3 (0.2-1.3 mg/dL), aspartate transaminase at 18 (15-46 U/L), alanine transaminase at 27 (13-69 U/L), and alkaline phosphatase at 71 (38-126 U/L). The patient was started on MMI 10 mg orally daily. Three weeks later, she developed sharp epigastric pain, diarrhea, lack of appetite, and fever. Her lipase was elevated to 1780 (23-300 U/mL) consistent with acute pancreatitis. Her triglycerides were normal at 81 (<200 mg/dL). She had no history of alcohol abuse. Abdominal computed tomography (CT) was consistent with acute uncomplicated pancreatitis, without evidence of gallstones or malignancy. It was thought that an upper endoscopic procedure performed days prior to her symptoms was the cause of her acute pancreatitis. However, in view of few reports of MMI-induced pancreatitis, MMI was discontinued, and her hyperthyroid symptoms were managed with propranolol. Her acute episode of pancreatitis quickly resolved clinically and biochemically (Figure 1).

One year later, she redeveloped mild symptoms of hyperthyroidism consisting of occasional anxiety, short-lived episodes of palpitations, and heat intolerance. Her thyroid function tests again showed a suppressed thyroid stimulating hormone of 0.02 (0.47-4.68 µIU/mL), a normal free T4 of 1.13 (0.78-2.19 ng/dL), and a normal free T3 of 3.7 (2.8-5.3 pg/mL). Her LFTs were normal, total bilirubin at 0.9 (0.2-1.3 mg/dL), aspartate transaminase at 24 (15-46 U/L), alanine transaminase at 20 (13-69 U/L), and alkaline phosphatase at 68 (38-126 U/L). Following a discussion of treatment alternatives with the patient, MMI 10 mg orally daily was restarted. Five days later, she developed progressive abdominal discomfort. Her lipase was elevated at 3512 (23-300 U/mL), and the abdominal CT showed inflammatory changes compatible with acute uncomplicated pancreatitis. As before, no evidence of gallstones or malignancy was identified. MMI was again discontinued, which was followed by rapid resolution of her pancreatitis (Figure 1). The patient is currently considering undergoing definitive therapy with radioactive iodine ablation.

## Discussion

Antithyroid drugs are used worldwide as the primary treatment of choice for hyperthyroidism, or as preparative therapy before more definitive therapies—radioiodine or surgery. The decision to use antithyroid drugs as primary treatment must be weighed against the risks and benefits, and the patient’s preference. Minor side effects of antithyroid drugs include skin reactions, arthralgias, and gastrointestinal effects, which occur in about 5% of patients. A less common but more detrimental side effect is agranulocytosis (an absolute granulocyte count of less than 500/mm^3^), with estimated frequency of 0.1% to 0.5%, and usually occurs within the first 2 months of treatment but may also occur years later. Hepatotoxicity is another major side effect, with estimated frequency of 0.1% to 0.2%, more common with propylthiouracil (PTU) than MMI. Liver enzymes do increase in untreated hyperthyroidism, and transient acute increases do develop following antithyroid treatment that eventually resolve while therapy is continued. Reactive vasculitis is the third major toxic reaction seen more commonly with PTU than MMI.^1^ Drug-induced pancreatitis (DIP) is a very rare side effect of MMI, and has only been described in case reports.^2-6^

DIP is usually based on the following 4 criteria: (*a*) development during the administration of the drug, (*b*) resolution after cessation of the offending drug, (*c*) recurrence after rechallenge of the suspected drug, and (*d*) exclusion of all other common causes.^7,8^ While over 525 different drugs can induce acute pancreatitis as an adverse reaction, according to a World Health Organization database, only 31 drugs have been associated with an established definite cause-and-effect relationship.^9^ The incidence of DIP is estimated to be up to 5.3%, which is still rare yet is the third most frequent cause of pancreatitis following cholelithiasis and alcoholism.^7^ However, the true incidence of DIP is difficult to assess as it is primarily based on case reports or small series. In addition, DIP is rarely accompanied by clinical or biochemical evidence of a drug reaction, such as rash, lymphadenopathy, or eosinophilia.^10^ Several other criteria developed and evolved to help define and classify DIP. In 1975, Karch and Lasagna used 5 terms (definite, probable, possible, conditional, and doubtful) to evaluate the probability of an adverse event to be caused by a suspected drug.^11^ In 2005, Trivedi and Pitchumoni formed 3 groups (classes I to III) to classify drugs reported to cause DIP based on presence of rechallenge.^12^ In 2007, Badalov et al created a similar criteria (classes 1-4) based on presence of rechallenge following an extensive review of existing published case reports.^13^ Considering the aforementioned classifications, MMI-induced pancreatitis in our case falls into “definitive” under Karch and Lasagna,^11^ “class III” under Trivedi and Pitchumoni,^12^ and “class 1b” under Badalov et al.^13^

Five cases of acute pancreatitis following MMI treatment have been previously reported.^2-6^ To our knowledge, ours is the sixth case. Summary of all 6 cases of acute pancreatitis following MMI treatment, including ours, is listed in Table 1.^2-6^ All 6 cases were adults, mostly females, and majority were Asians. The onset of MMI-induced pancreatitis was within 3 months of therapy. The time was even shorter on rechallenge. It occurs even with dose reduction and in the presence of modest doses of MMI (ie, 10 mg) daily. It is, therefore, unlikely to be dose-dependent. The mechanism of MMI-induced acute pancreatitis remains unclear. Possible theories proposed include pancreatic duct constriction, arteriolar thrombosis, hepatic involvement, accumulation of toxic metabolites, intermediary direct cellular toxicity, cytotoxic, osmotic, pressure, or metabolic effects,^14^ hypersensitivity reactions,^2^ and autoimmune reactions.^15,16^ Our patient is unique as she is the first case of MMI-induced pancreatitis occurring in the setting of toxic MNG. Autoimmunity is unlikely in our patient as she had negative thyroid-stimulating immunoglobulin, antithyroid peroxidase, and thyroglobulin antibodies antibodies. Cholestasis was not the mechanism as she had normal LFTs and normal abdominal CT. She also lacked physical findings of Graves’ disease such as ophthalmopathy or pretibial myxedema. In the presence of these severe side effects, one alternative may be to switch the patient to a different antithyroid medication. However, carbimazole (which is converted into MMI in the body) should likewise be avoided since one case of acute pancreatitis linked to carbimazole has been reported.^17^ On the other hand, no cross-reactivity has been found with PTU (Table 1), although the number of cases is too small to draw a definitive conclusion.

**Table 1.** Salient Features of the 6 Cases of MMI-Induced Pancreatitis.

Authors	Age	Gender	Ethnicity	Type of Hyperthyroidism	MMI Dose	Rechallenge	Interval Between Start of MMI and Development of Pancreatitis	Outcome
Taguchi et al^2^	66	Female	Japanese	Graves’ disease	1st: 30 mg; 2nd: 10 mg	+	1st: 3 weeks; 2nd: 3 hours	Switched to PTU and tolerated well
Su and Zou^3^	19	Female	Chinese	Graves’ disease	1st: 5 → 10 mg	−	2.5 months	RAI Tx
Abraham et al^4^	80	Female	Caucasian	Not specified	10 mg	−	3 months	Refused MMI rechallenge
Yang et al^5^	18	Female	Chinese	Graves’ disease	1st: 20 mg; 2nd: 10 mg; 3rd: 10 mg; 4th: 10 mg	+	1st: 4 days; 2nd: Few hours; 3rd: Few hours; 4th: Few hours	Switched to PTU and tolerated well
Jung et al^6^	51	Male	Korean	Graves’ disease	1st: 20 mg; 2nd: 10 mg	+	1st: 2 weeks; 2nd: 5 hours	Not specified
Agito and Manni (current case)	51	Female	Caucasian	MNG	1st: 10 mg; 2nd: 10 mg	+	1s: 3 weeks; 2nd: 5 days	Leaning toward RAI Tx

Abbreviations: MMI, methimazole; PTU, propylthiouracil; RAI, radioactive iodine; MNG, multinodular goiter.

In summary, our case as well as previous case reports in the literature should raise awareness about the possibility of pancreatitis in subjects treated with MMI even at low doses in the presence of suggestive symptoms. Appropriate laboratory investigation should be performed, and if the diagnosis is confirmed, MMI should be discontinued even in the presence of coexisting more common causes of pancreatitis.
